# Orthopædic Apparatus

**Published:** 1871

**Authors:** 


Orthopædic Apparatus.—We desire to call special attention to the card of D. W. Kolbe, Mechanist to the Philadelphia Hospital. Mr. Kolbe has recently issued a pamphlet descriptive of the Orthopædic Apparatus now most frequently employed in the treatment of deformities and deficiencies of the human body, with directions for taking measurements for their application.

This little pamphlet will be sent free, to any address, upon application.

From a personal acquaintance of several years with Mr. Kolbe, and a more extended acquaintance with his instruments and appliances, we with great pleasure reccommend him to the profession.

